# Accuracy of Dental Models Fabricated Using Recycled Poly-Lactic Acid

**DOI:** 10.3390/ma16072620

**Published:** 2023-03-25

**Authors:** Koudai Nagata, Keitaro Inaba, Katsuhiko Kimoto, Hiromasa Kawana

**Affiliations:** 1 Department of Oral and Maxillofacial Implantology, Kanagawa Dental University, 82 Inaoka-cho, Yokosuka 238-8580, Japan; 2 Department of Oral Microbiology, Kanagawa Dental University, 82 Inaoka-cho, Yokosuka 238-8580, Japan; 3 Department of Fixed Prosthodontics, Kanagawa Dental University, 82 Inaoka-cho, Yokosuka 238-8580, Japan

**Keywords:** material extrusion, 3D printer, poly-lactic acid, sustainable development goals, dental model, digital dentistry

## Abstract

Based on the hypothesis that the fabrication of dental models using fused deposition modeling and poly-lactic acid (PLA), followed by recycling and reusing, would reduce industrial waste, we aimed to compare the accuracies of virgin and recycled PLA models. The PLA models were recycled using a crusher and a filament-manufacturing machine. Virgin PLA was labeled R, and the first, second, and third recycles were labeled R1, R2, and R3, respectively. To determine the accuracies of the virgin and reused PLA models, identical provisional crowns were fitted, and marginal fits were obtained using micro-computed tomography. A marginal fit of 120 µm was deemed acceptable based on previous literature. The mesial, distal, buccal, and palatal centers were set at M, D, B, and P, respectively. The mean value of each measurement point was considered as the result. When comparing the accuracies of R and R1, R2, and R3, significant differences were noted between R and R3 at B, R and R2, R3 at P, and R and R3 at D (*p* < 0.05). No significant difference was observed at M. This study demonstrates that PLA can be recycled only once owing to accuracy limitations.

## 1. Introduction

Over the past few years, dental materials and equipment have evolved remarkably, benefiting both dentists and patients by improving the quality of treatments and reducing treatment times. When creating prosthetics such as crowns and bridges, professionals commonly take impressions after the formation of the abutment tooth or after building the abutment, injecting plaster, and creating a dental model. Impression taking dates back to the 1800s when wax and plaster were the most commonly used materials. However, non-reversible hydrocolloid alginate impression materials extracted from seaweed and reacted with gypsum to form insoluble calcium alginate have been used since the 1900s owing to their low costs and ease of use. These materials still represent the mainstay of dental treatments [1,2,3]. However, the poor dimensional stability of alginate impression materials when used alone for abutment teeth and the difficulty in reproducing margins have led to the applications of union impressions using alginate and agar for abutment teeth [4]. In the late 1900s, a silicone impression material was developed with vinyl polysiloxane as a component. In silicone impression materials, vinyl polysiloxane and polysiloxane hydroxide are additionally polymerized using platinum chloride to create a cross-linked structure and induce hardening [5]. Basapogu et al. [6] reported that the dimensional accuracy of silicone impression materials had an error ranging from 0.6% to 0.2%; however, the dimensional accuracy was better than that of alginate impression materials. Rajendran et al. [7] also performed silicone impressions on implant abutments. The authors reported on the usefulness of silicone impression materials for implant treatments. However, owing to cost and operability issues, impressions using alginate and agar are more commonly used, whereas silicone impression materials are only seldom used [8]. Aroma injection, a paste-type allied alginate impression material, has been developed recently. Chen et al. [9] reported that this material was more consistent than silicone impressions, had a lower contact angle than silicone, was more fluid, and allowed for more seamless impression taking than agar. Plaster models have also been used for dental models since the 1800s. Currently, ordinary gypsum, primarily composed of beta hemihydrate gypsum; hard plaster, primarily composed of alpha hemihydrate gypsum; and ultrahard plaster, are used for various purposes [10,11]. It was also used to record intermaxillary relationships and dental models [12]. For prosthetic dentistry, Taggart introduced the casting method in 1907, which is considered the foundation of current prosthetic treatments [13]. Vojdani et al. [14] reported a marginal fit of 88 ± 11 µm and an internal gap of 77 ± 10 µm for metal crowns cast and fabricated from wax patterns, demonstrating an excellent fit accuracy. Yang et al. reported a good marginal fit for a single metal coping produced by lost wax casting: 93 µm for a Ni–Cr alloy and 52 µm for a noble alloy [15]. Reitemeier et al. [16] reported a 20-year survival rate of 79% in 95 patients with 190 cast single crowns. Thus, dentistry has benefited from advances in materials science. The fabrication of prostheses and models using intraoral scanners (IOSs), computer-aided design/computer-aided manufacturing (CAD/CAM) systems, and 3D printers is now feasible [17]. The first IOS is believed to be the one launched by CEREC in 1985. IOSs use confocal, holographic, and shape-from-motion methods to illuminate the surface of an object with a laser, acquire three-dimensional data, and convert the data into polygon information, a set of triangular surfaces. This facilitates the reduced use of plaster casts, less discomfort during impression taking, and digital data storage [18]. It also reduces the risk of errors owing to the absence of plaster expansion and deformation of impression materials in conventional workflows [19]. Di Fiore et al. [20] compared eight IOSs, that is, True Definition, Trios, CEREC Omnicam, 3Dprogress, CS3500, CS3600, Planmeca Emerald, and Dental Wings, with regard to the accuracy of abutments and reported results of 31 ± 8 µm, 32 ± 5 µm, 71 ± 55 µm, 107 ± 28 µm, 61 ± 14 µm, 101 ± 38 µm, 344 ± 121 µm, and 148 ± 64 µm, respectively. In addition, as dentists primarily provide oral care, they are at an increased risk of infection from bodily fluids, aerosols, and droplet infections, such as the currently prevalent COVID-19 [21]. Papi et al. [22] noted that in the traditional workflow, impression materials with blood or saliva and plaster could be sources of infections. Therefore, they reported that the digital workflow, which only requires sterilization of IOS tips, reduces the risk of infection. Furthermore, Joda et al. [23] compared treatment times between IOSs and conventional silicone-based impression taking. They reported that the average working time for a student group was 5 ± 2 min using an IOS and 12 ± 2 min using the conventional method, whereas dentists reported a duration of 5 ± 1 min using an IOS and 10 ± 1 min using the conventional method; both groups had shorter treatment times using IOSs. The widespread use of CAD/CAM has also improved the quality of ceramics and zirconia, allowing for greater precision and a shorter time for crafting dental prosthetics [24,25]. With the advent of digital technology, dental treatments are becoming increasingly effective. Albuha Al-Mussawi et al. [26] mentioned that virtual reality simulators and augmented reality (AR) technology could be applied to dentistry for dental training, education, and the fabrication of technological objects. Furthermore, Ariwa et al. [27] evaluated the accuracy of digital dental models, namely head-mounted displays (HMDs) and spatial reality displays (SRDs), as reflected in AR devices. They reported that the measurement errors ranged from 0.3 to 2 mm for the HMDs and from 0.02 to 0.6 mm for the SRDs, indicating that the error was significantly higher for the SRDs than for the HMDs. Digitalization in dentistry is expected to accelerate further.

From the perspective of environmental issues, sustainable development goals are attracting attention worldwide. In this study, we focused on one of the targets of Goal 12, “Ensure sustainable consumption and production patterns”, which indicates that “by 2030, significantly reduce waste generation through prevention, reduction, recycling, and reuse”. Wayman et al. [28] reported that 359 million metric tons (Mt) of plastics were produced in 2018, of which an estimated 14.5 Mt entered the ocean, causing potential harm to host organisms consuming them. Consequently, growing concerns have been raised regarding environmental issues, and attempts are being made worldwide to reduce plastics, for instance, by charging for plastic bags and eliminating plastic straws [29,30]. Research is underway to degrade polyethylene terephthalate and polypropylene food and beverage packaging waste to address the long-term persistence of plastics in the environment [31]. We believe that using IOSs will reduce impression material applications in the future. Plaster models are often replaced by resin models sculpted using stereolithography 3D printers (SLA) and digital light processing (DLP). This is because they are generally considered to exhibit reasonable accuracy. Ishida et al. [32] created a cylindrical pattern mimicking a full crown and compared the material extrusion (MEX) and SLA. They claimed that SLA was more accurate and that MEX had a high surface roughness. They also mentioned the importance of 3D printer performance, as dental 3D printers have better accuracy than private ones. Resin is not recyclable; therefore, resin models can cause industrial waste. However, thermoplastic materials such as those used in MEX are recyclable. Therefore, we used one of the MEXs, fused deposition modeling (FDM) and polylactic acid (PLA) filaments. MEX is applied in medical devices, building structures, automobiles, and aerospace owing to its high printing strength, a wide range of available materials, and low cost per part [33]. However, the use of MEX and PLA to create dental models has not yet been reported in the literature. In a previous study, we reported on the accuracy of fit for PLA, resin, and plaster models [34]: 118 ± 22 μm, 62 ± 16 μm, 50 ± 27 μm for buccal areas; 64 ± 32 μm, 48 ± 24 μm, 76 ± 11 μm for palatal areas; 62 ± 28 μm, 50 ± 17 μm, 78 ± 20 μm for mesial areas; and 86 ± 43 μm, 50 ± 12 μm, and 80 ± 39 μm for distal areas, respectively, suggesting the usefulness of PLA models. PLA is a plant-derived plastic material that is expected to reduce carbon dioxide emissions. It is biodegradable and can dissociate into water and carbon dioxide in a compost environment [35]. PLA filaments can be reused owing to their characteristics [36]. We consider that using MEX and PLA to fabricate dental models, followed by their reuse, would reduce industrial waste. However, assessing the corresponding accuracy for applications in clinical practice is essential.

This study aimed to compare the accuracies of recycled PLA and virgin PLA models.

## 2. Materials and Methods

A left upper first molar model (A55A-262, NISSIN, Tokyo, Japan) was attached to a jaw model (Prosthetic Restoration Jaw Model D16FE-500A(GSE)-QF, NISSIN, Tokyo, Japan) as the base model. Impressions of the base models were taken using an IOS (Trios 3^®^; 3 shape, Copenhagen, Denmark), and resin blocks (ASAHI PMMA DISK TEMP; ASAHIROENTGEN IND. CO., LTD., Kyoto, Japan) were machined using CAD/CAM (Exocad^®^; Exocad, Berlin, Germany) (Ceramill motion2^®^; Amann Girrbach, Wien, Austria) based on the stereolithography (STL) data recorded to fabricate provisional crowns. Based on the manufacturer’s recommendations, the cement space was set to 0.11 mm, and the margin thickness was set to 0.06 mm. For the PLA model, impressions of the base models were taken using the IOS, and from the data obtained, PLA models were fabricated using 1.75 mm PLA filaments designed for Moment 3D printers (Moment Co., Ltd., Seoul, Republic of Korea) and MEX (Moment M350; Moment Co., Ltd., Seoul, Republic of Korea). Details regarding the filaments and MEX are summarized in Table 1.

**Figure 1:**
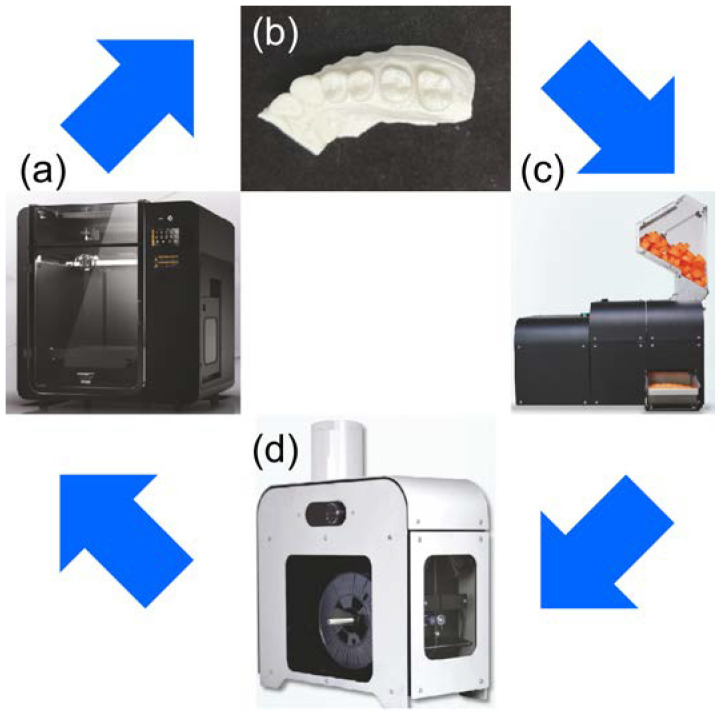
Process involved in poly-lactic acid (PLA) model recycling. (**a**) Moment M350 was used for MEX. (**b**) PLA models were prepared using 1.75 mm PLA filaments and MEX. (**c**) PLA models were ground using a filament-grinding machine (SHR3D IT). (**d**) After pulverization, filaments were produced again using a filament-manufacturing machine (COMPOSER).

**Figure 2:**
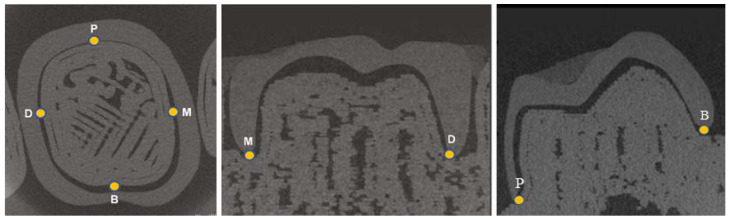
Provisional crown was placed on each model, and the marginal fit was measured using micro-computed tomography at the mesial center (M), distal center (D), buccal center (B), and palatal center (P) of the tooth.

In the recycling process, the PLA models were ground using a filament-grinding machine (SHR3D IT; 3devo B.V., Utrecht, The Netherlands), followed by filament production in a filament-making machine (COMPOSER; 3devo B.V). The manufactured filaments were used to fabricate the PLA models (Figure 1). The model made from virgin PLA was labeled R; PLA was recycled up to three times, and the first, second, and third PLA recycles were labeled as R1, R2, and R3, respectively. Five models for each type were fabricated, amounting to 20 in total. Following the manufacturer’s recommendations, the temperature during MEX was set to 225 °C, the lamination pitch was set to 100 μm, and the temperature of the filament-manufacturing machine was set to 170–190 °C. No models were surface treated, and no other materials were added when the filaments were reused. The marginal fits of the provisional crown and PLA model were used as accuracy measures. A PLA model with a provisional crown was placed perpendicular to the X-ray beam in a micro-computed tomography (CT) tube, and micro-CT (ScanXmate-L080T; Comscantecno Co., Ltd., Kanagawa, Japan) was used for imaging. The same provisional crown was placed on all the models. The occlusal surfaces of the provisional crown and adjacent teeth were fixed using utility wax (GC, Tokyo, Japan). The imaging conditions were as follows: 50 kV, 145 µA, voxel size of 34.5 μm, and magnification of 2.891×. After the images were recorded, the digital imaging and communications in medicine (DICOM) data were obtained for accuracy using a three-dimensional image analysis system volume analyzer (SYNAPSE VINCENT^®^, FUJIFILM, Tokyo, Japan). The measurement method included loading the DICOM data acquired by micro-CT into SYNAPSE VINCENT^®^, adjusting the contrast in the 3D viewer, selecting “linear measurement”, and determining the marginal fits of the provisional crown and PLA model. In total, four measurement points were set as the mesial center (M), distal center (D), buccal center (B), and palatal center (P) (Figure 2). The average value of each measurement point was used as the result.

The accuracy of the model was verified based on Dunnett’s test using the bell curve in Excel (Social Survey Research Information Co., Ltd., Tokyo, Japan). Continuous data were expressed as mean ± standard deviation. Differences with a *p*-value < 0.05 were considered statistically significant.

## 3. Results

The results of this study are summarized in Table 2. For R, the accuracies at positions B, P, M, and D were 68 ± 16 μm, 66 ± 22 μm, 88 ± 13 μm, and 60 ± 31 μm, respectively. For R1, the accuracies at positions B, P, M, and D were 76 ± 34 μm, 86 ± 23 μm, 72 ± 27 μm, and 50 ± 12 μm, respectively. For R2, the accuracies at positions B, P, M, and D were 86 ± 36 μm, 216 ± 99 μm, 78 ± 44 μm, and 78 ± 48 μm, respectively. For R3, the accuracies at positions B, P, M, and D were 154 ± 94 μm, 336 ± 77 μm, 132 ± 49 μm, and 132 ± 41 μm, respectively; thus, the accuracies for R and R1 were lower than 120 μm, and those for R2 and R3 were greater than 120 μm at all measurement points if standard deviations were included.

**Figure 3:**
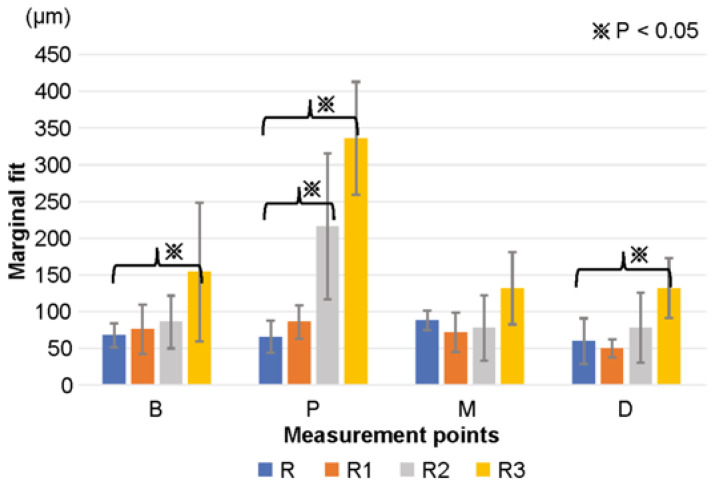
Mean and standard deviation of the marginal goodness of fit for each model and provisional crown were measured using SYNAPSE VINCENT^®^. The results for all models of R, R1, R2, and R3 are presented.

When comparing the accuracies of R with those of R1, R2, and R3, significant differences were noted between R and R3 at position B (*p* < 0.05), R and R2, R3 at position P (*p* < 0.01), and R and R3 at position D (*p* < 0.01) (Figure 3). A significant decrease was observed in the accuracy of R3.

## 4. Discussion

With the widespread use of IOSs and CAD/CAM, the fabrication of prostheses without model creation is now feasible. However, models are still essential for margin, contact, and occlusal adjustments. Numerous reports indicate that the marginal fit discrepancy of CAD/CAM crowns should be less than 120 μm [37,38,39]. However, the results of this study, including standard deviations, exceed 120 μm at all measurement points for R2 and R3. 

In MEX, the thermoplastic material is melted and extruded from a hot end to form a printed layer to produce the desired object [40,41]. Alsoufi et al. [42] reported that the shape error of PLA was within 3.00% on each side of a 40 mm (L) × 40 mm (W) × 15 mm (H) specimen, which is excellent accuracy for PLA fabricated by MEX. Only one PLA filament was used in this study. Cicala et al. [43] used MEX and three different commercial filaments to verify the accuracy using the same object. Two filaments that exhibited significant shear-thinning behavior and were correlated with mineral filler formulations printed well, but one had poor accuracy. Cicala et al. reported that differences in additives in the filament manufacturing process led to these accuracies. PLA is hydrolyzed during molding, which then degrades into low molecular weight oligomers. The oligomers further decompose into lactide and lactic acid, resulting in the loss of plastic properties. It has also been reported that when PLA is reused, the mechanical properties deteriorate because of hydrolysis and breakage of the reinforcing fibers [44,45]. Agüero et al. reported the following mechanical properties for reused PLA: impact strength (kJ·m^−2^) of 58 ± 4 for virgin PLA, 56 ± 4 after one recycle, and 36 ± 5 after four recycles. The elongation at break (%) was 10 ± 0.04 for virgin PLA, 9 ± 0.3 after two recycles, and 7 ± 0.9 after four recycles. The authors reported that the material could be recycled up to six times, with a slight degradation in the mechanical properties after one and two cycles but a marked decrease from the fourth cycle [46]. Zhao et al. also reused PLA and reported that the viscosity at 160 °C was approximately 2000 Pa·s for virgin PLA, approximately 750 Pa·s after the first cycle, and approximately 100 Pa·s after the second cycle; moreover, they reported that the viscosity decreased with repeated reuse, and the molecular weight decreased with chain scission, resulting in the degradation of mechanical properties. Therefore, they reported that reuse after the second cycle was difficult [47]. 

Anderson et al. compared the mechanical properties of virgin PLA and one-time reused PLA. They reported an 11% decrease in the tensile strength, a 7% increase in the shear strength, and a 2% decrease in the hardness of the reused filament, with no differences in the average mechanical properties of one-time reused PLA compared to those of the virgin material. However, they reported an increase in the standard deviation and greater variability in the results for the recycled material [48]. These reports are similar to our results. We believe that the mechanical properties of PLA degrade, and their stability is impaired the more they are reused, resulting in a higher standard deviation. As dental models only tolerate minimal errors in micrometer units, reusing them after the second cycle may be difficult. However, research is underway to add other materials to PLA to compensate for the PLA weaknesses. Beltrán et al. added a chain extender and an organic peroxide to PLA and evaluated its mechanical properties. They discovered that both additives reacted with terminal carboxyl groups in the aged polymer, causing cross-linking, branching, and chain extension reactions. Notably, both additives failed to improve either the viscosity or the thermal stability of the heavily degraded PLA. However, they reported that they could improve the microhardness of the recycled material [49]. Patwa et al. reported that adding 1 wt% crystalline silk nanodisks to a PLA matrix increased the toughness by approximately 65%, elongation by approximately 40%, and tensile strength by approximately 10% [50]. López et al. reported that mixing virgin PLA with 30 wt% recycled PLA and adding an epoxy-based chain extender and microcrystalline cellulose as reinforcements improved the tensile strength by up to 88%, modulus by 127%, and Izod impact strength by 11% [51]. Other studies have focused on adding materials such as metals, carbon, and fibers to PLA to maintain and improve its mechanical properties [52,53]. Furthermore, some studies involve reusing PLA with other materials [54,55]. Thus, research on reusing PLA and adding additives to maintain or improve its mechanical properties is progressing worldwide. The decrease in accuracy after the second recycle in this study could be attributed to the fact that the mechanical properties of PLA are known to deteriorate when reused.

Although minimal progress has been achieved in maintaining the biodegradability and mechanical properties of PLA, we believe it is possible to increase the number of recycling times for PLA, with improvements in the future. To the best of our knowledge, this study is the first to consider the reuse of PLA in dentistry.

PLA is widely used in the medical field, and numerous reports on its good biocompatibility can be found in the literature [56,57,58]. 

Concerning the use of PLA in dentistry, Benli et al. compared the marginal gaps of PLA, polymethyl methacrylate, and polyetheretherketone as provisional crowns. The results for PLA, polymethyl methacrylate, and polyetheretherketone were 60.40 ± 2.85 μm, 61 ± 4 μm, and 56 ± 5 μm, respectively, demonstrating the usefulness of PLA crowns [59]. Molinello–Mourelle et al. reported similarly on the usefulness of provisional crowns fabricated using PLA [60]. Crenn et al. examined the mechanical properties of PLA to verify its feasibility for use as provisional crowns. The elastic modulus of PLA is E = 3784 ± 99 MPa, that of nanoparticulate bisacryl resin is E = 3977 ± 878 MPa, and that of acrylic resin is E = 2382 ± 226 MPa. The flexural strength of PLA is Rm = 116 ± 2 MPa, that of nanoparticulate bisacryl resin is Rm = 86 ± 6 MPa, and that of acrylic resin is Rm = 115 ± 21 MPa, indicating mechanical property problems compared to the other two materials [61]. Relatively fewer reports have been presented on the application of PLA in dentistry, and most reports focus on its applications in provisional crowns. However, the glass transition temperature of PLA is known to be 50–80 °C [62,63]. PLA improves crystallinity and increases heat resistance. Notably, methods adopted to improve crystallinity include plasticizing modification and adding nucleating agents [64,65]. Among these, plasticizing modification is the most effective approach to improve crystallinity. However, the approach is reported to lower the glass transition temperature [66] simultaneously. Xu et al. reported that adding ethylene butyl methacrylate glycidyl methacrylate terpolymer and talc as nucleating agents for PLA increased the heat deformation temperature from 58 °C to 139 °C. The glass transition temperature, however, remained almost unchanged [67]. Various other heat resistance analyses have been conducted. However, no straightforward method has been identified to improve the definite glass transition temperature [68,69]. Additionally, while improving heat resistance in the future, impurities added to achieve heat resistance must be ensured not to impair the biodegradability of PLA [70]. Placing PLA crowns in the oral cavity is challenging due to heat resistance issues. Instead, we consider them more effective when used as models. 

PLA models are typically created using MEX. However, MEX is known to release volatile organic compounds (VOCs) during the molding process [71,72]. Ding et al. reported that the mass yields of VOCs emitted during MEX for PLA, acrylonitrile butadiene styrene, and polyvinyl alcohol were 0.03%, 0.21%, and 2%, respectively, at 220 °C [73]. Wojtyła et al. reported that the main VOC emitted from PLA was methyl methacrylate, which accounted for 44% of the total emissions. Thus, it is essential to keep the laboratory rooms unoccupied and ventilated during molding and restrict the use of several MEX processes simultaneously [74]. Notably, filaments left in an environment with 60–70% humidity for two weeks will degrade printing quality. Suharjanto et al. reported that filament storage using medium-density boards prevents and reduces air absorption of PLA filament and filament life, leading to the maintenance of the printing system. Note that the accuracy after modeling varies depending on the storage method [75].

In this study, we measured the marginal fit between the provisional crown and the model. However, it is necessary to measure the accuracy of the entire model in the future. Liu et al. examined the geometric accuracy of monkey tooth roots. After scanning the monkey’s maxilla with cone-beam CT and segmentation of the incisor roots, titanium implants were fabricated using laser powder bed fusion (PBF), a metal composite fabrication method. The extracted teeth and 3D-printed implants were scanned with a micro-CT and compared with the original segmented STL data. Results were reported as 91 ± 5% for the segmented versus printed tooth and 67 ± 11% for segmented versus actual. They found that monkey denticles are small and difficult to segment with high precision and that irregular shapes, surfaces, and technical challenges make it difficult to delineate regions of interest and cause deviation errors [76]. In the future, measuring the overall accuracy of the base model and the PLA model after molding will be necessary. This study has some limitations: the mechanical properties of PLA could not be verified, and PLA could not be investigated with additives. They will be the topic of future research. 

## 5. Conclusions

Sustainable development goals are attracting global attention in terms of environmental issues. Digital technology has led to improved accuracy in prosthetic treatment and shorter treatment times. However, SLA and DLP are widely employed in dentistry, and the resulting models are considered industrial waste. Therefore, we used MEX and PLA to reduce industrial waste in dentistry. Notably, PLA is a plant-derived plastic material that is expected to reduce carbon dioxide emissions. Further, biodegradable PLA filaments break down into water and carbon dioxide in a composting environment, and their properties allow them to be reused. This study examined the accuracy of MEX and PLA models in dentistry and the system for their reuse. The results show that PLA models made with MEX are within the acceptable range of 120 μm up to the first cycle and can be reused for up to one cycle. PLA may be considered the new material of choice in dentistry. The accuracy of MEX could be improved, and additives could be added to filaments to promote their reusability. This may reduce the industrial waste generated by dentistry.

## Figures and Tables

**Table 1:** Specifications of the filament and 3D printers used in this study.

	Specifications
PLA filament designed for Moment (Moment Co., Ltd., Seoul, Republic of Korea)	Material PLA: (>98%) Density: 1.25/cm Melting Point: 190 °C Recommended Print Temperature: 215–230 °C Thermal Distortion: 58 °C Water Absorption: 0.50% Molding shrinkage: 0.30
Moment M350 (Moment Co., Ltd., Seoul, Republic of Korea)	XYZ accuracy: XY: 12 μm, Z: 0.625 μm Laminating pitch: 0.05–0.3 mm Modeling size: 350 mm × 190 mm × 196 mm Nozzle: 0.4 mm

**Table 2:** Marginal fit results for virgin PLA and reused PLA models at each measurement point (μm).

	B	P	M	D
R	68 ± 16	66 ± 22	88 ± 13	60 ± 31
R1	76 ± 34	86 ± 23	72 ± 27	50 ± 12
R2	86 ± 36	216 ± 99	78 ± 44	78 ± 48
R3	154 ± 94	336 ± 77	132 ± 49	132 ± 41

B—buccal center; P—palatal center; M—mesial center; D—distal center.

## Data Availability

The data presented in this study are available on request from the corresponding author. The data are not publicly available due to ethical issues.
